# miR-422a inhibits osteosarcoma proliferation by targeting BCL2L2 and KRAS

**DOI:** 10.1042/BSR20170339

**Published:** 2018-03-21

**Authors:** Hao Zhang, Qian-Yun He, Guang-Chao Wang, Da-Ke Tong, Ren-Kai Wang, Wen-Bin Ding, Cheng Li, Qiang Wei, Chen Ding, Pei-Zhao Liu, Hao-Chen Cui, Xin Zhang, Di Li, Hao Tang, Fang Ji

**Affiliations:** 1 Department of Orthopedics Trauma, Changhai Hospital, Second Military Medical University, Shanghai, China; 2 Department of Oncology, NO.113 Hospital of People’s Liberation Army, Ningbo, China

**Keywords:** BCL2L2, KRAS, miR-422a, osteosarcoma

## Abstract

Osteosarcoma is the most common primary malignant bone tumor in children and adolescents. However, the underlying mechanism of osteosarcoma carcinogenesis and progression remains unknown. In the present study, we evaluated the expression profile of miRNAs in osteosarcoma tissues and the adjacent normal tissues. We found that the expression of miR-422a was down-regulated in osteosarcoma tissues and cell lines. In addition, we observed significantly elevated levels of repressive H3K9me3 and H3K27me3 and decreased active H3K4me3 on the promote region of miR-422a in osteosarcoma cells and clinical samples. Furthermore, up-regulation of miR-422a exhibited both *in vitro* and *in vivo* anti-tumor effects by inhibiting osteosarcoma cell growth and inducing apoptosis and cell cycle arrest. We also found that miR-422a targeted BCL2L2 and KRAS and negatively regulated their protein expression. Furthermore, restoration of miR-422a and knockdown of BCL2L2 and KRAS promoted apoptosis and induce cell cycle arrest in osteosarcoma cells. Taken together, the present study demonstrates that miR-422a may serve as a tumor suppressor in osteosarcoma via inhibiting BCL2L2 and KRAS translation both *in vitro* and *in vivo*. Therefore, miR-422a could be developed as a novel therapeutic target in osteosarcoma.

## Introduction

Osteosarcoma is the most common primary malignant bone tumor in children and adolescents, accounting for 30% of all bone malignancies and 3–4% of pediatric tumors [1]. In addition, osteosarcoma is associated with poor prognosis and high morbidity [2]. Despite surgical operation combining chemotherapy and radiotherapy, the five-year survival rate of patients with recurrent or metastatic osteosarcoma remains at ∼30% [3]. Thus, exploration of new targets is critical for developing novel therapeutic strategies for osteosarcoma patients.

MicroRNAs (miRNAs) are a group of endogenous non-coding RNAs with 18–25 nucleotides which negatively regulate gene expression by binding to the 3′untranslated region (UTR) of target mRNAs [4]. Accumulating studies have demonstrated that miRNAs play crucial roles in regulating diverse biological processes including cell proliferation, apoptosis, differentiation, and migration [5]. For example, Lei et al. have found that miRNA-145 inhibits osteosarcoma cell proliferation and invasion by targeting Rho-associated protein kinase 1 (ROCK1) [6]. Another study by Zhang et al has shown that miRNA-143, down-regulated in osteosarcoma, promotes apoptosis and suppresses tumorigenicity by targeting Bcl-2 [7]. Moreover, altered expression of individual miRNAs has been associated with prognosis, tumor stage, vascular invasion, and lymph node metastasis in multiple tumor types, including osteosarcoma [8]. Therefore, it is valuable to explore the miRNAs expression profile and further to identify specific miRNA involved in the osteosarcoma pathogenesis and progression.

DNA methylation-mediated epigenetic silencing is considered as an important mechanism of miRNA suppression in human cancer. It has been estimated that 10% of miRNAs are controlled by DNA methylation, with aberrant promoter methylation being responsible for miRNA dysregulation [9,10]. In the present study, we aimed to evaluate the miRNA expression profile in osteosarcoma and identify specific miRNA involving in the development and progression of osteosarcoma. Moreover, the mechanism responsible for the deregulation of miRNA in osteosarcoma was investigated.

## Materials and methods

### Patients

Tissue samples were obtained from patients pathologically diagnosed with osteosarcoma undergoing surgery at the department of Orthopedics Trauma of Changhai Hospital of Second Military Medical University (Shanghai, China). None of those patients had received chemotherapy or radiotherapy before the surgical excision. The present study was approved by the Medical Research Ethics Committee and informed consent was obtained prior to the present study. Tissue samples were stored at −80°C immediately for further investigation.

### Cell culture

Osteosarcoma cell lines, MG63 and U2OS, were purchased from American Type Culture Collection (ATCC, USA). They were cultured in DMEM with 10% FBS, 100 U/ml penicillin and 100 mg/ml streptomycin in a humidified atmosphere containing 5% CO_2_ at 37°C.

### Microarrays

RNA was isolated using TRIzol reagent according to the manufacturer’s protocol (Invitrogen, USA) and the concentration and purity of the samples were measured using a Nanodrop spectrophotometer. Labeling was performed using miRCURY™ Array power labeling kit. Hy3-labeled samples were mixed with Hy5-labeled reference RNA and hybridized to the miRCURY arrays. After hybridization, slides were scanned using GenePix 4000 Microarray Scanner System. Finally, significantly differentially expressed miRNA were identified using Prediction Analysis for Microarrays software.

### RNA extraction and real time PCR

Total RNA was extracted from the tissues and cells using TRIzol according to the manufacturer’s instructions (Invitrogen, USA). Reverse transcription PCR was performed with ReverTra Ace® kit (TOYOBO, Japan). Real time PCR was conducted using SYBR Premix Ex Taq (Takara, Japan). The miRNA-422a specific primer sequences were as follows: 5′-GTCGTATCCAGTGCAGGGACUGGAC UUAGGGUCAGAAGGCTCAGGAA -3′. The universal small nuclear RNA, U6, was used as an endogenous control. For each sample, independent experiments were repeated three times. The relative expression levels of mRNA and miRNA were analyzed by use of the 2-ΔΔCt method.

### Chromatin immunoprecipitation (ChIP)

ChIP assays were performed as previously described [11] by using EZ-ChIP Kit according to the manufacturer’s instructions (Millipore, USA). The dissociated DNA from immunoprecipitated protein/DNA complex was used for PCR assay.

### Transfection

MG63 and U2OS cells were seeded into 24-well plates and incubated overnight, then transfected with negative control RNA, miR-442a mimic or inhibitor using INTERFERin transfection reagent (Polyplus).

### Cell viability analysis

Cell viability was determined using the CCK-8 method. In brief, MG63 or U2OS cells were seeded into 96-well plates and cultured for the indicated time periods. Viable cells were counted by absorbance measurements at 450 nm by a microplate reader.

### Cell apoptosis and cell cycle analysis

For cell apoptosis analysis, cells at the density of 1.0 × 10^6^/ml were stained with Annexin V-FITC and PI for 15 min. For cell cycle analysis, prior to incubation with PI, cells were fixed in 75% cold ethanol at −20°C overnight. Cells were analyzed by a FACS Calibur flow cytometer (BD Biosciences) and data were analyzed with CellQuest software.

### Luciferase activity assay

Luciferase reporter plasmid containing the potential binding sequence of 3′UTR of BCL2L2 or KRAS mRNA or mutated sequence were co-transfected into HEK293 cells in 96-well plates with miR-422a mimic and the corresponding controls by using INTERFERin transfection reagent (Polyplus). Subsequently, firefly luciferase activity was determined and normalized to the corresponding Renilla luciferase activity 48 h after transfection.

### Animal model

Animal experiments in the present study were performed in compliance with the guidelines of the Institute for Laboratory Animal Research and consented by the Animal Ethics Committee. Six- to eight-week-old male athymic nude mice were anesthetized by exposure to 3% isoflurane and were subcutaneously injected with 5.0 × 10^5^ tumor cells. Subsequently, the tumor weight was measured once a week for 6 weeks.

### Western blot analysis

Osteosarcoma cells were lysed and protein concentrations were measured with BCA assay (Thermo Scientific, USA). For protein separation, equal amount of the extracts were subjected to SDS–PAGE and transferred onto nitrocellulose membranes. The membrane was then incubated with the primary antibodies (Cell Signaling Technology, USA) followed by incubation with the appropriate secondary antibodies. The protein bands were detected by ECL detection kit (Amersham).

### Statistical analysis

Data were presented as the mean ± standard deviation (SD) and analyzed by SPSS 17.0 (SPSS Inc., USA). When comparing two groups, Student’s *t*-test was used to calculate the differences. When comparing more than two groups, a one-way analysis of variance (ANOVA) was used followed by a LSD test. *P*<0.05 was considered as statistically significant.

## Results

### miR-422a was down-regulated in osteosarcoma tissues and cell lines

**Figure 1:**
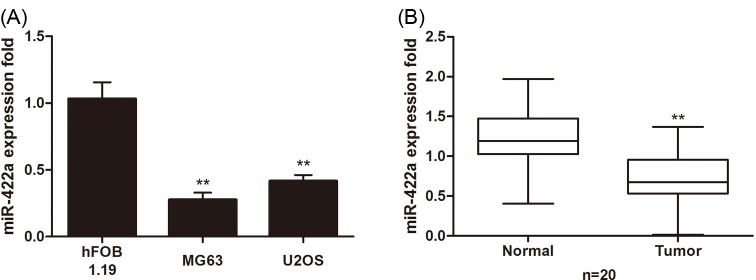
Down-regulation of miR-422a in osteosarcoma (**A**) Real time PCR was performed to measure the expression of miR-422a in osteosarcoma cell lines (MG63 and U2OS) and hFOB1.19 cells. (**B**) Twenty paired osteosarcoma tissues were collected, and the expression of miR-422a was detected using real time PCR. ** *P*<0.01.

Firstly, we discovered the miRNA expression pattern in osteosarcoma tissues and the adjacent normal tissues. miRNA microarray analysis identified many differentially expressed miRNA (see Supplementary Material). Among them, we focused on the role of miR-422a in osteosarcoma because it was seldom reported. The fold change of miR-422a (microarray data) in osteosarcoma tissue compared to normal tissue was 0.2137. Moreover, we evaluated the expression of miR-422a in human osteoblast hFOB1.19 cells and osteosarcoma cell lines including MG63 and U2OS. Real time PCR showed that miR-422a was obviously down-regulated in osteosarcoma cell lines compared to hFOB1.19 cells (Figure 1A). Moreover, miR-422a was also decreased in 20 paired osteosarcoma tissues compared to their matched normal tissues (Figure 1B). Collectively, these results suggested that miR-422a may play an important role in osteosarcoma pathogenesis.

### Altered histone modification on miR-422a promoter in osteosarcoma

**Figure 2:**
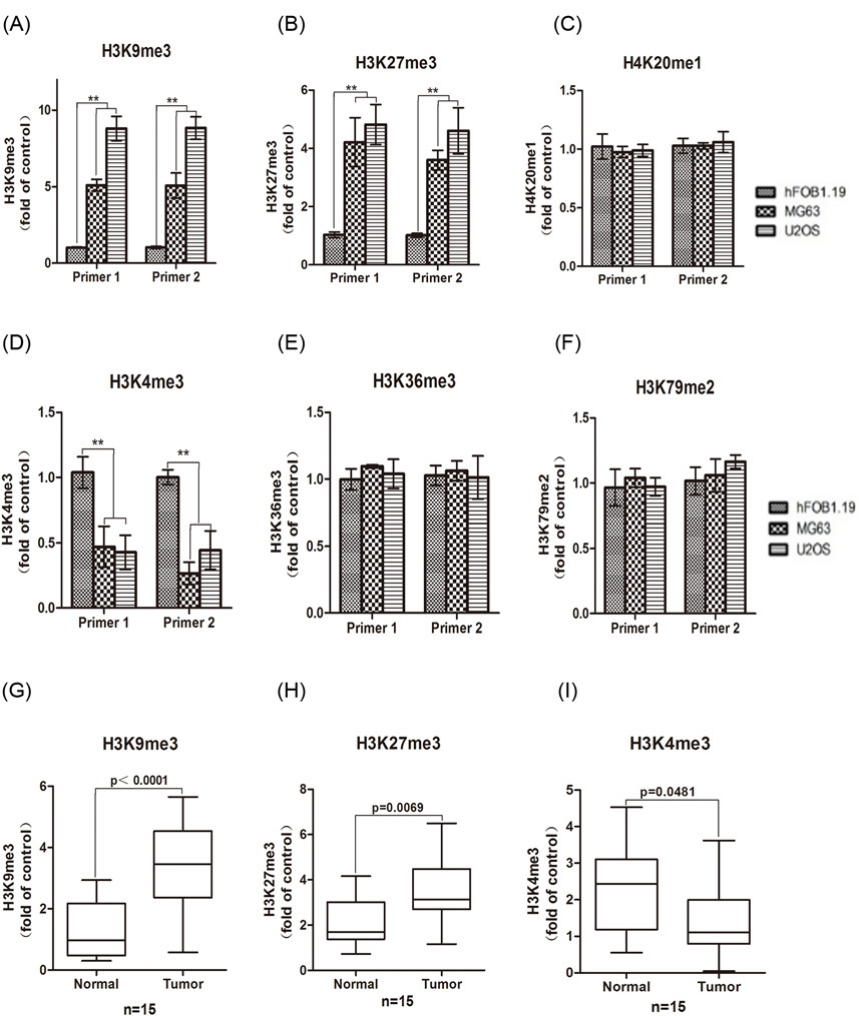
Deregulated histone modification on miR-422a promoter ChIP assay was performed in MG63, U2OS and hFOB1.19 cells using the antibodies H3K9me3 (**A**), H3K27me3 (**B**), H4K20me1 (**C**), H3K4me3 (**D**), H3K36me3 (**E**), and H3K79me2 (**F**) followed by real time PCR. Fifteen paired osteosarcoma tissues were collected and ChIP assay was conducted using the antibodies H3K9me3 (**G**), H3K27me3 (**I**), and H3K4me3 (**H**) followed by real time PCR. ** *P*<0.01.

Next, we investigated the mechanism of down-regulation of miR-422a in osteosarcoma. We detected the methylation status of permissive modification marks (H3K4me3, H3K36me3, and H3K79me2) and repressive histone modification marks (H3K9me3, H3K27me3, and H4K20me1) on miR-422a upstream promoter. ChIP and real time PCR analysis showed that, compared to those marks in hFOB1.19 cells, we observed significantly elevated levels of repressive H3K9me3 and H3K27me3 modification (Figure 2A,B) and decreased active H3K4me3 modification (Figure 2D). On the contrary, we detected no obvious differences in H4K20me1, H3K36me3, and H3K79me2 levels (Figure 2C,E,F).

Furthermore, we evaluated these histone modification marks on miR-422a promoter region in 15 paired osteosarcoma samples. Similarly, we detected increased levels of H3K9me3 and H3K27me3, and decreased H3K4me3 in cancerous tissue compared to the adjacent normal tissues (Figure 2G–I). Collectively, these data suggested that inhibition of permissive histone modification and elevation of repressive histone modification may contribute to down-regulation of miR-422a in osteosarcoma.

### miR-422a inhibits osteosarcoma cell proliferation *in vitro* and *in vivo*

**Figure 3:**
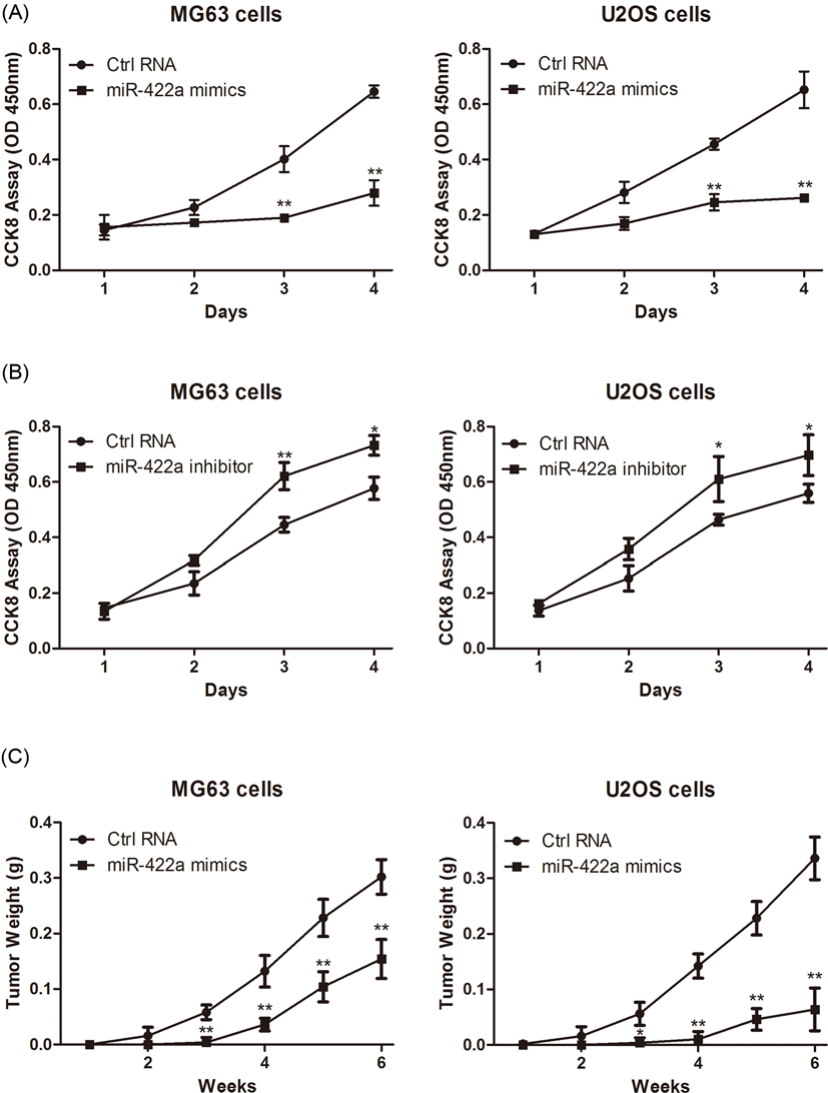
miR-422a inhibits osteosarcoma cell growth *in vitro* and *in vivo* MG63 and U2OS cells were transfected with miR-422a mimic (**A**) or inhibitor (**B**) followed by CCK-8 assay to measure cell viability at different time points. (**C**) Nude mice were injected with 5.0 × 10^5^ tumor cells transfected with miR-422a mimic or control, and the tumor weight was measured once a week for 6 weeks (at least six mice in each group). * *P*<0.05; ** *P*<0.01.

In order to explore the role of miR-422a in osteosarcoma cell proliferation, we transfected miR-422a mimic or inhibitor into MG63 and U2OS cells. Then, cell viability was detected at different time points after transfection with miR-422a mimic or inhibitor. Consequently, CCK-8 assay showed that up-regulation of miR-422a inhibited the proliferation of MG63 (Figure 3A, left) and U2OS cells (Figure 3A, right). Conversely, inhibition of miR-422a led to enhanced proliferation of MG63 (Figure 3B, left) and U2 osteosarcoma (Figure 3B, right).

Furthermore, an *in vivo* model was applied to explore the role of miR-422a on tumorigenicity. As a result, we found that miR-422a transfected osteosarcoma MG63 and U2OS cells exhibited a delayed tumor formation compared to their negative controls (Figure 3C). Taken together, these data indicated that miR-422a inhibited the proliferation of osteosarcoma cells both *in vitro* and *in vivo*.

### miR-422a induces cell apoptosis and cell cycle arrest in osteosarcoma

**Figure 4:**
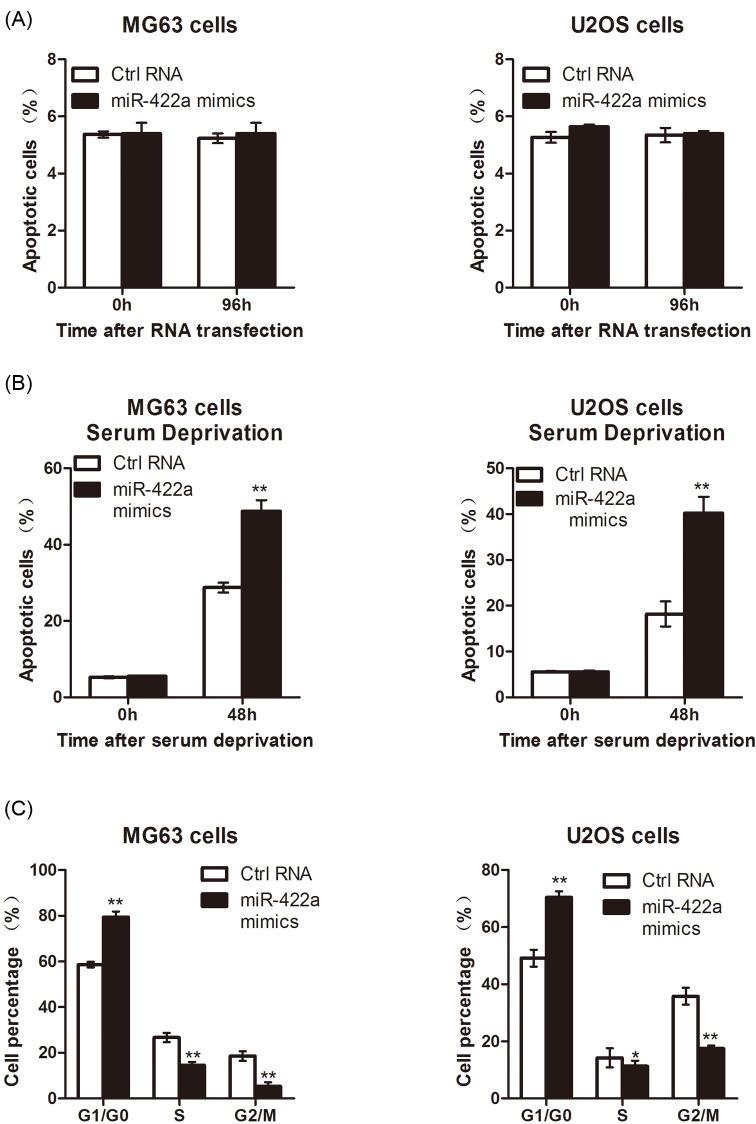
miR-422a induces osteosarcoma cell apoptosis and cell cycle arrest MG63 and U2OS cells were transfected with miR-422a mimic or negative control. After transfection, flow cytometry analysis was performed in these cells under normal condition (**A**) or serum deprived condition (**B**). In addition, cell cycle distribution was determined in MG63 and U2OS cells transfected with miR-422a mimic or negative control (**C**). * *P*<0.05; ** *P*<0.01.

Then, miR-422a mimic-transfected osteosarcoma cells were subject to flow cytometry for determination of apoptosis and cell cycle distribution. Consequently, Annexin V/PI staining showed that up-regulation of miR-422a had little effects on osteosarcoma cell apoptosis (Figure 4A). However, we found that enforced expression of miR-422a increased the apoptotic rate of MG63 and U2OS cells under serum deprived condition (Figure 4B). Moreover, we observed increased cell number in the G0/G1 phase while the cell number in the S and G2/M phases decreased after transfection with miR-422a mimic (Figure 4C). Collectively, these data suggested that elevated miR-422a promoted apoptosis under starvation conditions in osteosarcoma cells and induced cell cycle arrest in G0/G1 phase, thereby inhibiting the proliferation of tumor cells.

### miR-422a targeted BCL2L2 and KRAS in osteosarcoma cells

**Figure 5:**
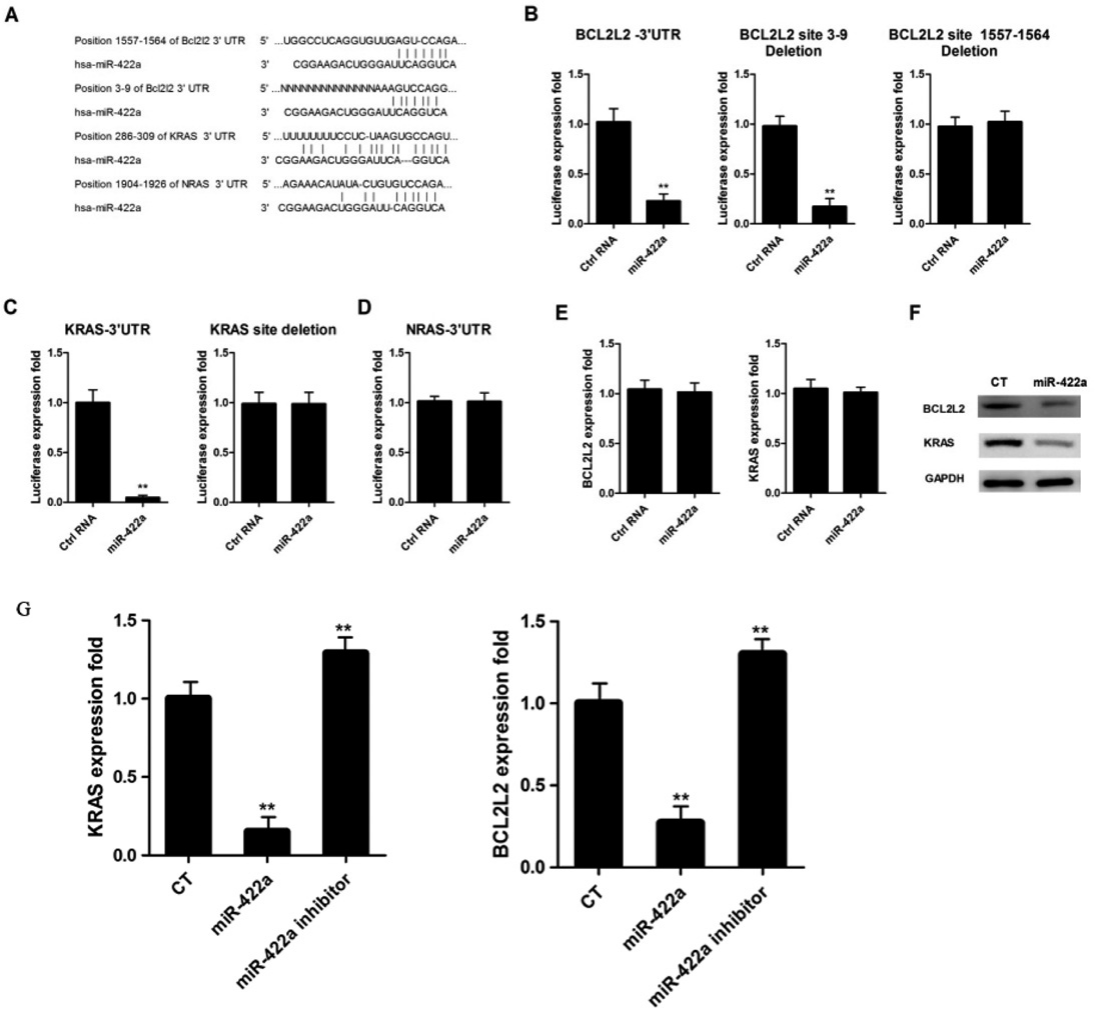
miR-422a targeted BCL2L2 and KRAS (**A**) Prediction of the targets of miR-422a. Dual-luciferase reporter assay was performed to validate that BCL2L2 (**B**) and KRAS (**C**) were direct targets of miR-422a, and NRAS was not (**D**). Effects of up-regulation of miR-422a on the mRNA (**E**) and protein (**F**) expression of BCL2L2 and KRAS. (**G**) Effects of inhibition of miR-422a on the protein expression of BCL2L2 and KRAS. ** *P*<0.01.

Given that miR-422a is involved in regulating cell proliferation and apoptosis, we subsequently predicted its targets which are related to apoptosis and proliferation using the online software miRecords (http://mirecords.biolead.org/). As a result, the 3′UTR of BCL2L2, KRAS, and NRAS mRNA had potential binding sites for miR-422a (Figure 5A). Then, the 3′UTR sequences of these three genes were inserted into pMIR vector and the luciferase activity was determined using the dual-luciferase reporter assay. Results showed that miR-422a mimic significantly inhibited luciferase activities of the reporter plasmid containing the wild-type BCL2L2 3′UTR (Figure 5B, left) and the mutated sequence in BCL2L2 3′UTR (site 3–9 deletion) (Figure 5B, middle), but without obvious changes in the reporter plasmid containing the mutated sequence in BCL2L2 3′UTR (site 1557–1564 deletion) (Figure 5B, right). In addition, miR-422a mimic remarkably decreased luciferase activities of the reporter plasmid containing the wild-type KRAS 3′UTR, but without significant changes in the reporter plasmid containing mutated KRAS 3′UTR (Figure 5C). However, miR-422a mimic had no effects on luciferase activities of the reporter plasmid containing the NRAS 3′UTR (Figure 5D). These data suggest that miR-422a directly targeted BCL2L2 at site 1557-1564 and KRAS at site 286-309.

Moreover, we found that up-regulation of miR-422a decreased the protein expression of BCL2L2 and KRAS (Figure 5F), but without obvious changes in the mRNA levels of BCL2L2 and KRAS (Figure 5E), and inhibition of miR-422a elevated the protein levels of BCL2L2 and KRAS (Figure 5G). Taken together, these data demonstrated that miR-422a exhibited its anti-tumor effects probably through inhibiting the protein expression of BCL2L2 and KRAS in osteosarcoma cells.

### Down-regulated miR-422a promoted osteosarcoma proliferation via increasing BCL2L2 and KRAS

**Figure 6:**
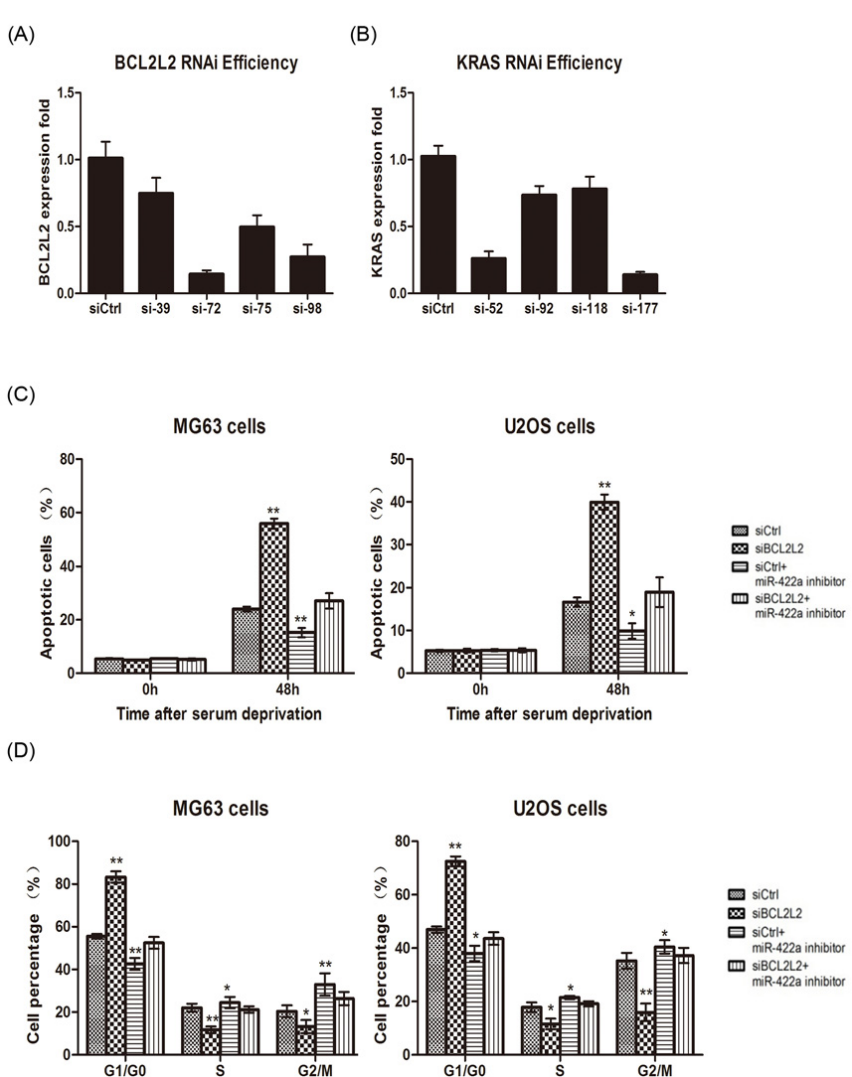
Down-regulated miR-422a promoted osteosarcoma proliferation via increasing BCL2L2 and KRAS Selection of siRNA for BCL2L2 (**A**) and KRAS (**B**). For apoptosis determination, MG63 and U2OS cells were transfected with si-BLC2L2 or miR-422a inhibitor alone or si-BLC2L2 combined with miR-422a inhibitor followed by flow cytometry analysis (**C**). Furthermore, cells were transfected with si-KRAS or miR-422a inhibitor alone or si-KRAS combined with miR-422a inhibitor, and the cell cycle distribution was analyzed. * *P*<0.05; ** *P*<0.01.

In order to ascertain the anti-tumor property of miR-422a is mediated by inhibition of BCL2L2 and KRAS, four siRNA specific for BCL2L2 (si-39, si-72, si-75, si-98) and KRAS (si-52, si-92, si-118, si-177) were generated using siRNA Selection software. Consequently, BCL2L2-si-72 (Figure 6A) and KRAS-si-177 (Figure 6B) presented the highest interference efficiency for BCL2L2 and KRAS, respectively, which were chosen for further investigation.

Subsequently, MG69 or U2OS cells were transfected with si-BLC2L2 or miR-422a inhibitor alone or si-BLC2L2 combined with miR-422a inhibitor. We found that transfection with si-BCL2L2 led to more apoptotic cells (Figure 6C). Meanwhile, inhibition of miR-422a resulted in less apoptotic cells, which was reversed by co-transfection with miR-422a inhibitor and si-BCL2L2 (Figure 6C). In addition, interfering KRAS expression significantly increased cell number in G0/G1 phase and decreased cell number in S and G2/M phases (Figure 6D). Transfection with miR-422a inhibitor led to decreased cell population in G0/G1 phase and more cells in S and G2/M phases, which was reversed by co-transfection with miR-422a inhibitor and si-KRAS (Figure 6D). Taken together, these results demonstrated that down-regulated miR-422a promoted the pathogenesis of osteosarcoma by increasing the expression of its target genes BCL2L2 and KRAS.

## Discussion

Osteosarcoma is a frequently occurred bone tumor characterized by an aggressive clinical course [12,13]. Recent studies have paid much attention to investigate the molecular mechanisms contributing to osteosarcoma carcinogenesis and progression. Increasing evidence suggests that altered expression of miRNA is critically important in the malignant behaviors of human cancers [14,15]. Thus, identification and investigation of the deregulated miRNAs in osteosarcoma development may provide novel therapeutic targets for osteosarcoma treatment.

In the present study, we applied miRNA microarray approach to examine the miRNA expression profile in osteosarcoma. As a result, we found a significant decrease of miR-422a in osteosarcoma tissues compared to the adjacent normal tissues. There have been several studies on the importance of miR-422a in human cancers. For example, a previous study has suggested that miR-422a is decreased in colorectal tumor and may exhibit a protective role against colon cancer [16]. However, another study showed that the expression of miR-422a is significantly higher in patients with multiple sclerosis, suggesting that miR-422a may have a deleterious effect on multiple sclerosis [17]. Recently, a clinical investigation has demonstrated that miR-422a has been reported to serve as an independent prognostic factor and function as a potential tumor suppressor in colorectal cancer [18]. In our study, the expression of miR-422a was remarkably decreased in osteosarcoma MG63 and U2OS cells compared to human osteoblast hFOB1.19 cells. Additionally, the down-regulation of miR-422a was also ascertained in 20 paired osteosarcoma tissues.

Although several studies have report the deregulated expression of miR-422a, little is known about the molecular mechanisms regulating miR-422a. In eukaryotic cells, histone acetylation has been shown to be critical for transcriptional activation of genes [19,20]. Histone hyperacetylation is linked to active transcription whereas histone hypoacetylation is associated with transcriptionally silent chromatin [21,22]. In the present study, we evaluated the methylation status of permissive modification marks (H3K4me3, H3K36me3, and H3K79me2) and repressive histone modification marks (H3K9me3, H3K27me3, and H4K20me1) on the promote region of miR-422a. ChIP analysis showed that repressive H3K9me3 and H3K27me3 were significantly elevated while the active H3K4me3 was inhibited both in osteosarcoma cells and clinical samples, suggesting that inhibition of permissive histone modification and elevation of repressive histone modification contributed to the decreased levels of miR-422a in osteosarcoma.

Furthermore, to identify the biological role of miR-422a, we transfected miR-422a mimic or inhibitor into MG63 and U2OS cells. Consequently, enforced expression of miR-422a inhibited osteosarcoma cell proliferation; while, inhibition of miR-422a promoted the growth of tumor cells. In addition, an *in vivo* model also demonstrated that miR-422a exhibited an anti-cancer effect by delaying the tumor formation ability. In addition, we determined the effects of miR-422a on osteosarcoma cell apoptosis and cell cycle distribution by performing flow cytometry analysis. Consequently, we found that enforced expression of miR-422a promoted apoptosis and induced G0/G1-phase cell cycle arrest, and thereby exhibited the anti-proliferative effects on osteosarcoma cells.

It is well known that miRNAs regulate tumor cell growth, apoptosis, differentiation, and migration via inhibiting the expression of target genes [23,24]. In order to identify miR-422a targets, we performed bioinformatics analysis and found three genes (BCL2L2, KRAS, and NRAS) possessing the potential binding sites at the 3′UTR. To confirm this prediction, luciferase activity assay was performed; results showed that up-regulation of miR-422a suppressed luciferase activities of the reporter plasmids containing both BCL2L2 and KRAS, but not NRAS. In addition, miR-422a decreased the protein expression of BCL2L2 and KRAS while the mRNA levels remained unchanged, suggesting that miR-422a exhibited the anti-tumor effect via regulating BCL2L2 and KRAS protein expression in osteosarcoma cells.

Accumulating evidence suggests that the proteins of the Bcl-2 family are critical for the intrinsic apoptotic pathway [25,26]. Cytochrome *c* is released by the mitochondria into the cytosol during apoptosis. Bcl-2 maintains the mitochondrial membrane integrity to prevent the release of cytochrome *c* [27]. As a member of the Bcl-2 family, BCL2L2 is similar to its close relative Bcl-2, and is involved in the carcinogenesis and progression in human cancers [28,29]. RAS is a small gene family known for its ability to induce carcinogenesis [30]. Three RAS genes, termed as HRAS, KRAS, and NRAS, have been identified in the mammalian genome. Increasing evidence suggests that the abnormal expression of miRNA and KRAS has been associated with tumorigenesis [31,32]. Therefore, we explored the relationship between miR-422a and its targets BCL2L2 and KRAS in osteosarcoma. We found that knockdown of BCL2L2 alone promoted apoptosis. Meanwhile, inhibition of miR-422a alone lead to decreased apoptotic cells, which was reversed by knockdown of miR-422a and BCL2L2. In addition, KRAS knockdown alone increased cell cycle arrest; but miR-422a inhibition promoted cell cycle progression, which was reversed by down-regulation of miR-422a and KRAS. Therefore, the modulation of BCL2L2 and KRAS by miR-422a may explain why the down-regulation of miR-422a during osteosarcoma carcinogenesis can promote cancer progression.

In conclusion, the present study demonstrates that deregulated histone modification is responsible for down-regulation of miR-422a in osteosarcoma. We also provide evidence about the *in vitro* and *in vivo* biological effects of overexpression of miR-422a in osteosarcoma. In addition, our results demonstrate that miR-422a functions as a tumor suppressor by directly targeting BCL2L2 and KRAS. Therefore, our study suggests that miR-422a may serve as a novel therapeutic target for osteosarcoma treatment.

## Supporting information

**Table:** 

## Abbreviations

ChIP: chromatin immunoprecipitation
miRNA: microRNA
UTR: untranslated region
